# Health System Response to the 2023 Floods in Emilia-Romagna, Italy: A Field Report

**DOI:** 10.1017/S1049023X23006404

**Published:** 2023-12

**Authors:** Martina Valente, Maicol Zanellati, Giulia Facci, Nicola Zanna, Emilio Petrone, Erika Moretti, Francesco Barone-Adesi, Luca Ragazzoni

**Affiliations:** 1. CRIMEDIM - Center for Research and Training in Disaster Medicine, Humanitarian Aid and Global Health, Università del Piemonte Orientale, 28100, Novara, Italy; 2. Department for Sustainable Development and Ecological Transition, Università del Piemonte Orientale, 13100, Vercelli, Italy; 3. Azienda AUSL Ferrara, Ferrara, Emilia-Romagna, Italy; 4. Department of Translational Medicine, Università del Piemonte Orientale, 28100, Novara, Italy

**Keywords:** disaster response, Emilia-Romagna, extreme weather event, floods, health system

## Abstract

In May 2023, the Italian region Emilia-Romagna was hit by intense rainfall, which caused extensive floods in densely populated areas. On May 4, 2023, a 12-month state of emergency was declared in the region with the activation of response and recovery plans. This field report provides an overview of the health response to the floods, paying particular attention to the measures put in place to ensure care for displaced populations and raising interesting points of discussion regarding the role of the health system during extreme weather events (EWEs). The considerations that emerge from this report underline the need for a primary care approach to disasters, especially when these occur in areas with a high prevalence of elderly resident population, and underscore the importance of integration of different levels of care.

## Specific Event Identifiers

- Event Type: Floods, Landslides
- Event Onset Date: May 2, 2023
- Location of Event: 80 Municipalities in the Provinces of Forlì-Cesena, Ravenna and in the Metropolitan Area of Bologna, within the Emilia-Romagna Region, Italy
- Geographic Coordinates: Latitude = 44.3640607; Longitude = 12.0590095
- Dates of Observation Reported: May 2, 2023 - June 15, 2023
- Response Type: Public Health Response

## Introduction

Italy has 93.4% of its municipalities at risk of floods, landslides, and coastal erosion.^1^ Emilia-Romagna is the region with the largest area at risk of floods due to a complex network of drainage canals and minor watercourses covering morphologically depressed areas.^2^ On May 2-3, May 9-10, and May 16-17, 2023, the region experienced torrential rains which caused extensive floods and landslides.

**Figure 1.**
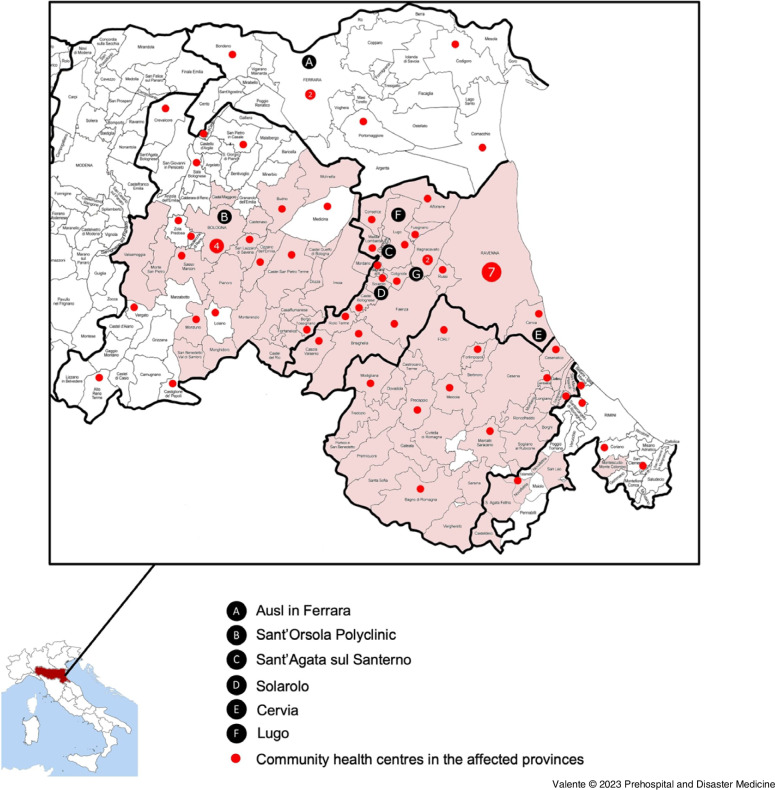
Map Indicating Affected Areas and Key Locations Mentioned in the Report.

Initial weather alerts were issued as early as April 20, 2023.^3^ On April 24-25, 2023, episodic thunderstorms and hail hit the provinces of Ferrara, Parma, Reggio Emilia, and Rimini.^4^ The heaviest rainfall began on May 2, 2023 and led to the activation of a red alert from the civil protection.^5^ In 48 hours, the rainfall event became the most intense in the region since 1997, resulting in the precipitation of over 200 millimeters of water in several areas of the Bologna and Forlì-Cesena provinces.^6^ On May 9-10, 2023, there was a second episode of intense rainfall with further accumulation of water due to soil saturation.^7^ Between May 15-17, 2023, precipitation reached 300 millimeters in Forlì, 150-200 millimeters in Ravenna and Bologna, and 150 millimeters in the Forlì-Cesena area.^8,9^ The precipitation observed in the first-half of May was up to eight-times higher than the reference climatological monthly average.^10^ By May 25, 2023, a total of 23 rivers overflowed and 13 reached threatening water levels, and over 370 major landslides took place in the region causing the closure of more than 700 roads,^11,12^ overall affecting an area hosting one million people^13^ (Figure 1).

## Objective

The objective of this field report is to provide an overview of the health response to the 2023 floods in Emilia-Romagna (Italy), paying particular attention to the measures put in place to ensure care for displaced populations and raising interesting points of discussion regarding the role of the health system during extreme weather events (EWEs).

## Data and Information Sources

This field report is based on three main data sources: (1) information provided in the After Action Report from the Crisis Unit of the local health unit (Azienda Unità Sanitaria Locale; AUSL) in the province of Ferrara; (2) verbal and written exchanges with health professionals, as well as provincial and regional key stakeholders involved in the operations; and (3) analysis of relevant documents and reports, such as ordinances for flood emergency management, produced by regional authorities and the civil protection in the aftermath of the disaster.

## Limitations and Strengths

This field report was developed in an early post-disaster phase and is therefore based on data immediately available to the research team and perishable information provided by first responders and key stakeholders involved in the health operations. Although the majority of information reviewed to develop this field report refers to the Emilia-Romagna region, most of the primary data collected in the context of consultations and exchanges with key stakeholders refer to the province of Ferrara.

A strength of the field report is that it is a result of a collaboration between an academic institution with expertise in Disaster Medicine and health actors working on the front line during the emergency. It is meant to be the starting point for a more comprehensive assessment of the health response to flooding, which will include a more in-depth analysis of health records when these are accessible and made available by local health authorities.

## Observations

### Declaration of State of Emergency and Activation of Response Plan

On May 3, 2023, the national civil protection service was activated in support of Emilia-Romagna.^14^. On May 4, 2023, a 12-month state of emergency in the areas impacted by the floods was declared.^15^ The civil protection has been active since the beginning to support the management of the floods with fire and rescue corps, local police, army, alpine rescue teams, and volunteers. The European Union activated the Civil Protection Mechanism, with Slovakia, Slovenia, and France offering support through their pumping teams.^16,17^ On May 22, 2023, there were 7,749 rescuers with 731 vehicles, 130 rescue boats, and 13 helicopters.^17^ Coordination was handled locally by the Municipal Operations Centers.

In the Ferrara area, on May 17, 2023, the general director of the AUSL established the provincial Crisis Unit (Figure 1, A). Following the notification of the potential evacuation of the hospital in Ravenna, the Disaster Manager in the Ferrara province was activated to establish reception centers.

### Evacuation and Displacement of People

The number of people evacuated from their homes grew exponentially since the first days of flooding. On May 3, 2023, up to 370 people were evacuated in Faenza, Castel Bolognese, and Conselice.^18^ On May 17, 2023, the evacuated people were 10,000, one-half of them located in gyms and hotels set up as reception centers by the municipality.^11^ On May 20, 2023, the number of people who had to leave their homes reached 36,600, of which 5,000 were hosted in reception centers.^19^ Trends of evacuated people started decreasing from May 21, 2023.^20^

In the Ferrara area, the Disaster Manager was initially in charge of establishing reception centers in two gyms hosting 38 and 48 people, respectively. Subsequently, a third reception center with capacity for 100 people was established following the notification of possible flooding in Lavezzola. The Italian Red Cross (Rome, Italy) started the census of all displaced people in the area on May 19, 2023.

### Health Emergency Management and Response Strategy

Health-related measures implemented in response to the floods concern four main domains.

*Domain 1: Postponement of Elective Visits and Deferral of Non-Urgent Services*—Elective visits were cancelled in the most affected areas. Non-urgent surgical operations were postponed until after May 24, 2023, and specialist out-patient services and collection of reports were only guaranteed for urgent cases. Emergency care, including surgery and non-deferrable treatments, was always guaranteed, even in the affected areas.^21^ New patients who had to undergo heart surgery were diverted to the Sant’Orsola Polyclinic in Bologna^22^ (Figure 1, B).

*Domain 2: Provision of Care for Displaced People and Emergency Medical Services—*General practitioners (GPs) periodically visited reception centers. Medicines usually paid for out-of-pocket were distributed free-of-charge.^23^ Mobile ambulatory services called PASS (Socio-Sanitary Assistance Posts) offering nursing and medical services were deployed in the municipalities of Sant’Agata sul Santerno and Solarolo^24^ (Figure 1, C and D). Psychological support was offered to displaced populations by over 120 psychologists specialized in emergency mental care. Psychological service was subsequently expanded to the whole population, with priority given to fragile categories.^25^

In the Ferrara province, the Crisis Unit activated extra family and community nurses that provided primary health care to people displaced, alongside GPs who voluntarily manifested interest in supporting.

With regard to Emergency Medical Services, the region relied on seven rescue coordination centers and 160 municipal operations centers. Because the operations center for emergency services experienced a 300% increase in incoming calls during the floods,^26^ Emergency Medical Services had to also rely on ambulances offered by non-governmental organizations.^27,28^ The local Emergency Medical Services deployed four helicopters in the areas of Pavullo, Parma, Bologna, and Ravenna.^29^ In cases of adverse weather conditions, rescue operations were carried out by alpine rescue volunteers specialized in aquatic environments.^30^

*Domain 3: Monitoring of Infrastructural Safety and Enhancement of Structural Surge Capacity—*Health infrastructures did not suffer major damage but were constantly monitored. Some first-aid points were temporarily closed, such as the one in Cervia and the emergency department in Lugo (Figure 1, E and F), with patients being redirected to Ravenna.^31^ The night between May 18-19, 2023, the Maria Cecilia Hospital (Figure 1, G), a private hospital specialized in cardiac surgery, was evacuated with 180 patients relocated to other hospitals, including 16 intensive care patients transferred through ambulances and helicopters.^32^ Hospitals and private clinics in less affected areas offered full availability of beds for redistributing patients.

*Domain 4: Implementation of Disaster-Related Public Health Strategies—*Livestock, wild animals, and fishes died, contributing to the deterioration of water quality because of high organic load and shortage of oxygen.^33^ To deal with issues deriving from possible outbreaks of infectious diseases, public health authorities recommended a booster of tetanus toxoid for people carrying out activities in flooded buildings, as well as those with serious injuries. Health institutions started infectious disease monitoring for gastrointestinal, cutaneous, and respiratory infections more frequently related to exposure to flood waters, or to living in damaged buildings or in conditions of overcrowding. On May 26, 2023, public health authorities released a leaflet with instructions on how to prevent infections in flooded areas. To avoid endangering the population, some but not all mass-gathering events were cancelled in the impacted areas.^34^

### Management of Vulnerable Categories

For fragile individuals, medically-equipped helicopters were activated for evacuation, available social houses were assigned to socioeconomically disadvantaged families, and a map was developed for helicopters to be able to supply isolated communities with essential medicines.^35^

In the Ferrara province, while at first all displaced people were indiscriminately housed in the same reception centers, from May 18, 2023, special structures were dedicated to elderly people, those with reduced mobility and disabilities, and those with multimorbidity. Geriatric triage was activated, where a specialist was in charge of assessing the degree of autonomy of individuals and whether they could stay in the equipped or normal reception centers. Equipped centers had special beds and facilitated access to toilets. If individuals required more advanced care, they were referred, alongside their caregiver, to community health centers, which were alerted and strengthened (Figure 1).

## Analysis

Typically after sudden-onset disasters, there is a peak in acute care needs due to trauma or injury.^36^ During floods, this peak is unusual, while the need to ensure continuity of chronic care for the population arises early on.^37^ A recent analysis examining the quality of life in Italy shows that Ferrara is one of the Italian provinces with the highest consumption of drugs for chronic diseases, particularly among the elderly.^38^ This suggests a high prevalence of people in need of continuous access to chronic care and underlines the importance of a primary care approach to disasters.

In Emilia-Romagna, successful strategies to guarantee continuity of care were the provision of free medicines and periodic visits from GPs in reception centers, as recommended by the scientific literature.^39^ Geriatric triage proved to be useful in enabling differentiation of assistance pathways and customized care. This was enhanced by the region’s long-standing focus on strengthening primary and home care, and promoting the integration of health care and social services.^40^ In fact, Emilia-Romagna could count on a particularly advanced network of 128 operational community health centers, that is 25.6% of all such facilities in Italy^41^ (Figure 1).

Effective response to disasters requires integration of primary care with public health functions and social services.^42^ In Emilia-Romagna, partnerships with private providers of medical disposals were established to allow citizens to access the necessary supplies.^43^ Public health functions were integrated across all levels of the health system to control infections and mitigate risks. In particular, guidelines emphasized the need for infectious disease surveillance at the primary care and prehospital level.^34^ Since the emergency was characterized by many needs of a social rather than medical nature, the setting up of a non-medical emergency number to meet all needs that do not require the dispatch of a rescue vehicle has been proposed.^44^

The health response to the Emilia-Romagna floods allowed the evacuation of a large number of people and the provision of continuous primary care for most. However, the health impacts are not limited to the weeks following the floods, but can extend to months and years after the event. This is particularly relevant when it comes to psychological distress^45,46^ and the secondary surge in primary care needs.^47^ As for the Emilia-Romagna floods, it is recommended that psychological support is maintained in the coming months, and that home assistance is strengthened for the chronically ill who had seen their health checks cancelled or postponed during the floods.

## Conclusion

Due to climate change, EWEs like floods are likely to increase and occur in countries with a long life-expectancy and an on-going process of population ageing, like Italy. This field report underlines the need for health systems to be prepared for responding to EWEs, not only through emergency health interventions, but also by predicting disasters’ medium- and long-term social and health impacts.
